# Two new species of genus *Ateleute* Förster (Hymenoptera, Ichneumonidae, Cryptinae) with a key to the Oriental species

**DOI:** 10.3897/zookeys.141.1912

**Published:** 2011-10-28

**Authors:** Mao-Ling Sheng, Gavin R. Broad, Shu-Ping Sun

**Affiliations:** 1 General Station of Forest Pest Management, State Forestry Administration, 58 Huanghe North Street, Shenyang 110034, P. R. China; 2 Department of Entomology, Natural History Museum, Cromwell Road, London SW7 5BD, UK

**Keywords:** Phygadeuontini, *Ateleute*, new species, Key, Oriental Region, taxonomy

## Abstract

Three species of *Ateleute* Förster 1869 belonging to the tribe Cryptini of the subfamily Cryptinae (Hymenoptera, Ichneumonidae), collected from Jiangxi Province, China, are reported, of which two are new for science: *Ateleute ferruginea* Sheng, Broad & Sun, **sp. n.** and *AAteleute zixiensis* Sheng, Broad & Sun, **sp. n.** One, *AAteleute densistriata* (Uchida, 1955), was previously known from China and Japan. A key to the species of genus *Ateleute* known in the Oriental Region is provided.

## Introduction

*Ateleute* Förster 1869, belonging to the subfamily Cryptinae of Ichneumonidae (Hymenoptera), comprises 33 described species (Yu et al. 2005), of which 3 are known from the Oriental, 3 from the Palearctic, 1 from the Nearctic (Townes 1967, also in the Neotropics), 3 from the Neotropical and 24 from the Ethiopian Region.

The species distributed in Ethiopian Region were reported by Seyrig (1952) and catalogued by Townes and Townes (1973). Kasparyan and Hernandez (2001) described two new species and one new subspecies and provided a key to the species known in the Neotropical Region. Four species are known in Japan (Ashmead 1906, Momoi 1970, Uchida 1955). The European and Oriental species were catalogued by Gupta (1987). So far, one species, *Ateleute densistriata* (Uchida, 1955), is known in Taiwan, China.

In the last four years the authors have been exploring Jiangxi Province, situated near the northern border of the Oriental part of China, and have collected large numbers of ichneumonids. In this article, the species belonging to *Ateleute*, collected in Jiangxi Province, P.R. China, are reported.

## Materials and methods

Specimens were collected using entomological sweep nets in the forests of Anfu, Ji’an, Longnan, Qianshan, Quannan and Zixi Counties, Jiangxi Province (CHINA).

Images of whole bodies were taken using a CANON Power Shot A650 IS. Other images were taken using a Cool SNAP 3CCD attached to a Zeiss Discovery V8 Stereomicroscope and captured with QCapture Pro version 5.1.

The morphological terminology is mostly that of Gauld (1991). Wing vein nomenclature is based on Ross (1936) and the terminology on (Mason (1986, 1990).

The new species were checked against the species described by Seyrig (1952) from Madagascar by the provided key and original descriptions.

Type specimens are deposited in the Insect Museum, General Station of Forest Pest Management (GSFPM), State Forestry Administration, People’s Republic of China.

### Ateleute

Förster, 1869

http://species-id.net/wiki/Ateleute

Ateleute  Förster, 1869. Verhandlungen des Naturhistorischen Vereins der Preussischen Rheinlande und Westfalens, 25(1868): 171. Type-species: *Ateleute linearis* Förster.
Psychostenus  Uchida, 1955: 32. Type-species: *Psychostenus minusculae* Uchida.
Talorga  Cameron, 1911: 63. Type-species: *Talorga spinipes* Cameron; monobasic.
Tsirirella  Seyrig, 1952: 44. Type-species: *Tsirirella tsiriria* Seyrig; designated by Townes, Townes and Gupta 1961.

#### Diagnosis.

 *Ateleute* can be distinguished from all other genera of Cryptini by combination of the following characters: Apical margin of clypeus sharp, truncate or concave. Lower tooth of mandible as long as or slightly longer than upper tooth. Epomia absent. Genal carina joining base of mandible or joining hypostomal carina near base of mandible. Sternaulus weak and shallow. Posterior transverse carina of mesosternum complete. Areolet large, receiving 2m-cu basal of its middle, 3rs-m absent or almost absent. Hind wing vein M+Cu strongly arched, 2-1A absent or very short. First tergum without median dorsal carina, often with longitudinal wrinkles, spiracle near its middle. Ovipositor sheath about 0.65 times as long as hind tibia. Tip of ovipositor distinctly elongate.

#### Host.

 According to previous records (Momoi 1977, Townes 1967, Uchida 1955), hosts are *Cryptothelea minuscula* Butler and *Astala confederata* (Grote & Robinson) (Psychidae).

##### Key to species known in Oriental region

**Table:** 

1	Female	2
–	Male	5
2	Forewing vein 1cu-a distinctly distal of 1/M. Forewing vein 2m-cu situated approximately at middle of areolet. Propodeum blackish brown. (Japan: Okinawa)	*Astala mesorufa* Momoi
–	Forewing vein 1cu-a at or slightly distal of 1/M. Forewing vein 2m-cu situated at basal 0.3 of areolet. Propodeum reddish brown	3
3	Median terga or apical terga black. Terga with or without white	4
–	All terga entirely brown, without white spot. (Male unknown). (China: Jiangxi)	*Astala ferruginea* Sheng, Broad & Sun, sp.n.
4	Tergum 1 approximately 1.7 times as long as apical width. Vertex entirely black. Scape and pedicel white or yellowish white. Terga 7 and 8 with white spots. (China: Jiangxi, Taiwan; Japan: Okinawa)	*Astala densistriata* (Uchida)
–	Tergum 1 approximately 2.6 times as long as apical width. Vertex black, with lateral white spots. Scape and pedicel brownish black entirely. Terga 7 and 8 entirely black. (Male unknown). (China: Jiangxi)	*Astala zixiensis* Sheng, Broad & Sun, sp.n.
5	Fore wing vein 2m-cu approximately at middle of areolet. Gonosquama very slender, apex pointed	*Astala mesorufa* Momoi
–	Fore wing vein 2m-cu distinctly before middle of areolet. Gonosquama broad	6
6	Propodeum with fine and dense punctures. Dorsolateral carina of first tergum complete. Body dark brown (Figure 13). (Female unknown). (Malaysia)	*Astala spinipes* (Cameron)
–	Propodeum with fine leathery texture. Dorsolateral carina of first tergum between spiracle and apex absent. Body black (Figure 11)	*Astala densistriata* (Uchida)

### Ateleute ferruginea

Sheng, Broad & Sun sp.n.

urn:lsid:zoobank.org:act:34931ABC-632E-4AC7-B3CD-44D8BE899142

http://species-id.net/wiki/Ateleute_ferruginea

Figures 1–5 

#### Etymology.

 The specific name is derived from the terga being entirely brown.

#### Types.

 *Holotype*, Female, CHINA: Shuangjiang Forest Farm, Ji’an County, 174 m, Jiangxi Province, 10 May 2009, leg. Lin-Da Li. Paratypes: 2 females, same data as holotype except 24 May 2009.

#### Diagnosis.

 *Ateleute ferruginea* can be distinguished from all other species of *Ateleute* by combination of the second tergum having circular, concentric striations (Fig. 5) and the following colour pattern: mesopleuron, mesosternum, propodeum, legs and metasoma brown to reddish brown; lateral portions of vertex and upper portion of inner orbits broadly white.

#### Description.

 Female. Body length 5.5 to 6.0 mm. Fore wing length 4.0 to 4.5 mm. Antenna length 6.0 to 6.5 mm. Ovipositor sheath length 1.5 to 1.8 mm.

#### Head.

 Face (Figure 2) 1.4 to 1.5 times as wide as long, lateral margins almost parallel, with fine granulose texture, median portion weakly convex. Clypeal suture weak. Clypeus evenly convex, with texture as that of face; median section of apical margin almost truncate, slightly concave centrally. Mandible and cheek with fine leathery texture. Mandible short, upper tooth as long as lower tooth. Malar space approximately 0.6 times as long as basal width of mandible. Gena very short, with fine leathery texture, strongly convergent backwardly. Vertex and frons with texture as that of gena. Posterior portion of vertex from hind margin of interocellar area to occipital carina slanted almost vertically, distinctly concave medially. Postocellar line slightly shorter than ocular-ocellar line. Frons almost flat, lower portion nearby antennal socket concave. Antenna longer than body, with 28 to 30 flagellomeres, median portion slightly thickened, ventral profile slightly flat, suddenly shortened from fifth flagellomere (Figure 3). Ratio of length from first to seventh flagellomeres: 8.0:7.9:7.7:6.9:5.6:4.0:3.7. Occipital carina dorso-medially interrupted.

#### Mesosoma.

 Pronotum, mesoscutum and dorsal and ventro-posterior portions of mesopleuron with fine granulose texture. Anterior margin of pronotum with indistinct fine longitudinal wrinkles; lateral concavity with indistinct and short transverse wrinkles. Mesoscutum slightly convex, median portion with dense oblique longitudinal wrinkles. Notaulus evident, reaching about 0.7 the distance to posterior margin of mesoscutum. Scutellum weakly convex, with texture as that of mesoscutum, but relatively finer than that; subapical portion with indistinct transverse concavity; lateral carina reaching to the concavity. Postscutellum weakly convex, posterior margin with fine carina-shaped edge. Median portion of mesopleuron (Figure 4) shallow oblique transverse concave. Sternaulus reaching middle coxa, posterior portion weak. Mesopleural fovea indistinct. Posterior transverse carina of mesosternum complete. Metapleuron with dense and unclear granulose texture. Subbasal portion of submetapleural carina triangularly convex, posterior end vanishing. Wings hyaline. Fore wing vein 1cu-a slightly distal of 1/M. Areolet pentagonal, vein 3rs-m disappearing, receiving 2m-cu at its basal 0.3 to 0.4. 2m-cu inclivous, with one bulla. Hind wing vein 1-cu approximately as long as cu-a, cu-a strongly reclivous. Legs slender. Dorsal profiles of tibiae and ventral profiles of tarsomeres with irregular and short spines, the spines separated by more than a spine length. Dorsal apex of hind first trochanter distinctly projecting. Ratio of length of hind first to fifth tarsomeres 5.7:2.4:1.5:0.6:1.0. Claws small. Propodeum with weak and indistinct fine transverse wrinkles. Area petiolaris with weak longitudinal wrinkles. Posterior transverse carina, pleural carinae and lateral carinae of area petiolaris present. Propodeal spiracle small, circular, approximately located basal 0.35.

#### Metasoma.

 First tergum 2.1 to 2.2 times as long as apical width, evenly and strongly narrowed toward base, median portion strongly arched, with dense, even and fine longitudinal wrinkles, dorsolateral carinae indistinct, ventrolateral carinae complete. Spiracle located slightly anterior of middle. Second tergum trapeziform, median portion with fine arcuate to circular lines (Figure 5), lateral with fine longitudinal wrinkles. Remaining terga with slightly fine leathery texture. Basal portion of third tergum with fine and weak transverse lines. Ovipositor sheath 0.5 to 0.6 times as long as hind tibia, apex truncated. Apical portion of ovipositor gradually pointed.

#### Color.

**Figures 1–5.**
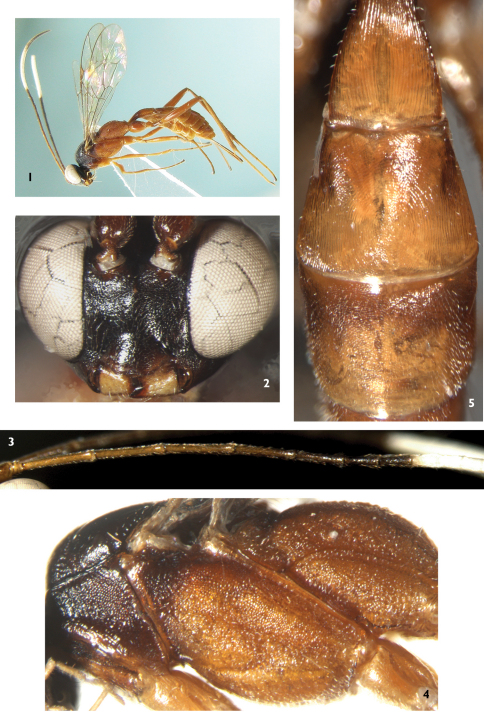
*Ateleute ferruginea* Sheng, Broad & Sun, sp.n. Holotype. Female **1** Body, lateral view **2** Head, anterior view **3** Basal portion of flagella **4** Mesosoma, lateral view **5** Terga 1 to 3.

 (Figure 1) Main body and legs brown to reddish brown, except the following: head, dorsal portion of pronotum and mesoscutum black. Scape, pedicel and flagellomeres 1 to 3 (4) dark brown. Flagellomeres 5 to 7 and apical portion black; 8 to 17 (18) white. Mandible except black teeth, maxillary and labial palpus yellow. Lateral portions of vertex and upper portion of frontal orbits broadly white. Lower portion of pronotum and scutellum darkish brown. Tegula brown. Stigma yellowish brown. Veins brownish black.

#### Remarks.

 This new species is similar to *Ateleute mesorufa* Momoi, 1970, but can be distinguished from it by the following combination of characters: areolet receiving 2m-cu at its basal 0.3 to 0.4; scape and pedicel dark brown; all terga entirely brown. *Ateleute mesorufa*: areolet receiving 2m-cu approximately at its middle; scape and pedicel white; apical portions of terga 1 to 3 and spot of tergum 6 white.

### Ateleute zixiensis

Sheng, Broad & Sun sp. n.

urn:lsid:zoobank.org:act:8D66A40C-9179-4143-B038-AAC08A4FE2B4

http://species-id.net/wiki/Ateleute_zixiensis

Figures 6–10 

#### Etymology.

 The specific name is derived from the locality of type.

#### Types.

 *Holotype*, Female, CHINA: Zixi County, 174 m, Jiangxi Province, 24 July 2009, leg. Mei-Juan Lou.

#### Diagnosis.

 *Ateleute zixiensis* can be distinguished from all other species of *Ateleute* by combination of: malar space approximately 0.9 times as long as basal width of mandible; first tergum 2.6 times as long as apical width; gena, vertex and frons almost smooth and shining; basal-median portion of second tergum (Figure 10) with fine and almost transverse lines, lateral portion with fine oblique longitudinal lines and the pronotum and main portion of terga black.

#### Description.

 Female. Body length about 7.5 mm. Fore wing length about 4.7 mm. Antenna length about 7.8 mm. Ovipositor sheath length about 2.0 mm.

#### Head.

 With fine granulose texture. Face (Figure 7) 1.6 times as wide as long, almost flat, with irregular indistinct transverse wrinkles; median portion convex. Clypeal suture weak. Clypeus approximately 1.5 times as wide as long, strongly convex, with sparse and fine punctures; median section of apical margin almost truncate, slightly concave centrally. Mandible short, with fine leathery texture; upper tooth slightly shorter than lower tooth. Malar space approximately 0.9 times as long as basal width of mandible. Gena, vertex and frons almost smooth and shining. Gena very short, strongly convergent backwardly. Posterior portion of vertex, from posterior ocelli to occipital carina, slanted almost vertically. Postocellar line approximately as long as ocular-ocellar line. Upper half of frons almost flat; lower half concave, with fine longitudinal wrinkles. Antenna filiform, with 32 flagellomeres, slightly thickened beyond middle; from middle to subapex with ventral profile slightly flat; suddenly shortened from fifth flagellomere (Figure 8). Ratio of length from first to seventh flagellomeres: 10.0:9.3:9.2:8.0:5.9:4.5:4.2. Occipital carina dorso-medially interrupted widely.

#### Mesosoma.

 Pronotum with irregular fine granulose texture; anterior and upper-median portions with longitudinal wrinkles, lower-posterior portion with oblique longitudinal wrinkles. Mesoscutum smooth, with distinct fine leathery texture; median portion rough, with oblique transverse wrinkles. Notaulus evident, reaching about 0.7 the distance to posterior margin of mesoscutum. Scutellum evenly convex, with texture as that of mesoscutum; lateral carina reaching to middle. Postscutellum small, rough, posterior portion with fine and weak transverse edge. Upper portion of mesopleuron (Figure 9) with fine granulose texture; upper anterior portion, under subalar prominence, with oblique transverse wrinkles; Remaining portion rough. Epicnemial carina laterally present on lower half of mesopleuron. Mesopleural fovea vestigial. Median portion of sternaulus present vestigially. Posterior transverse carina of mesosternum complete. Metapleuron rough. Submetapleural carina complete. Wings grayish hyaline. Fore wing vein 1cu-a opposite 1/M. Areolet pentagonal, vein 3rs-m disappearing, receiving 2m-cu at its basal 0.4. 2m-cu straight, inclivous, with one bulla. Hind wing vein 1-cu longer than cu-a, cu-a strongly reclivous. Legs slender. Dorsal apex of hind first trochanter distinctly projecting. Hind tibia except inner side and tarsomeres with short spines, spines on tibia separated by more than a spine length, distance between spines on tarsomeres as long as or shorter than a spine length. Hind fifth tarsomere more or less depressed. Ratio of length of hind first to fifth tarsomeres 10.0:5.0:2.7:1.2:2.0. Claws very small. Propodeum rough, dorsal profile with weak, fine and indistinct transverse wrinkles. Posterior transverse carina and pleural carinae present and strong. Propodeal spiracle small, circular, approximately located at basal 0.3.

#### Metasoma.

 First tergum 2.6 times as long as apical width, strongly narrowed toward base, smooth, with dense and fine longitudinal wrinkles. Basal and apical end of dorsolateral carina vestigially present. Ventrolateral carinae complete. Spiracle located slightly before middle of first tergum. Second tergum (Figure 10) rough, basal-median portion with fine and almost transverse lines, lateral portion with fine oblique longitudinal lines. Third tergum with fine granulose texture. Remaining terga almost shining. Fourth and fifth terga with indistinct transverse lines. Ovipositor sheath approximately 0.65 times as long as hind tibia, apex truncate. Apical portion of ovipositor gradually pointed.

#### Color.

**Figures 6–10.**
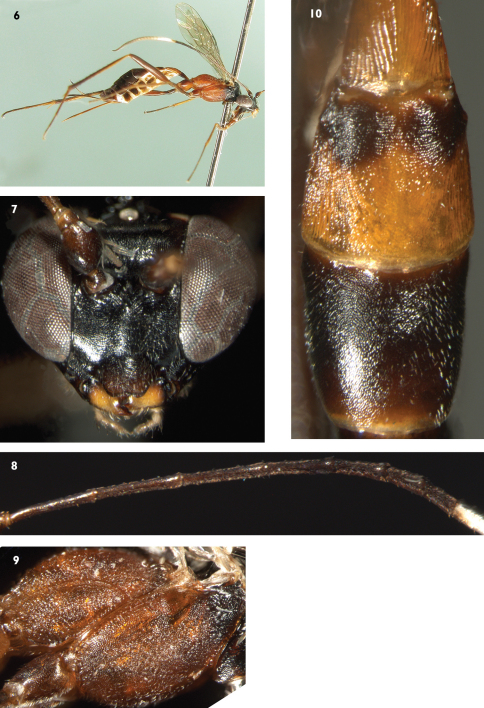
*Ateleute zixiensis* Sheng, Broad & Sun, sp.n. Holotype. Female **6** Body, lateral view **7** Head, anterior view **8** Basal portion of flagella **9** Mesosoma, lateral view **10** Terga 1 to 3.

 (Figure 6) Black, except the following. Scape, pedicel and flagellomeres 1 to 3 brownish black. Flagellomeres 8 to 12 white. Lateral spots of vertex and median portions of mandibles yellowish white. Labial palpus, basal portion and ventral profiles of hind tibia buff. Maxillary palpus, tegula, front and middle legs and hind tarsomeres dark brown. Ventral and dorsal profiles of hind coxa, hind trochanter and femur blackish brown. Mesopleuron except black upper-anterior portion, mesosternum, metanotum, metapleuron, postscutellum and propodeum reddish brown. First tergum except subbasal portion more or less blackish brown, second tergum except large brownish black spot, hind narrow margin of third tergum yellowish brown. Third and the following terga brownish black. Stigma and veins brownish black.

#### Remarks.

 This new species is similar in colour to *Ateleute ferruginea* Sheng, Broad & Sun, but can be distinguished from the latter by the following combination of characters: malar space approximately 0.9 times as long as basal width of mandible; first tergum 2.6 times as long as apical width; third and the following terga brownish black (Figure 6). *Ateleute ferruginea*: malar space approximately 0.6 times as long as basal width of mandible; first tergum 2.1 to 2.2 times as long as apical width; terga entirely brown to reddish brown (Figure 1).

### Ateleute densistriata

(Uchida, 1955)

http://species-id.net/Ateleute_densistriata

Figures 11–12

Psychostenus densistriatus  Uchida, 1955: 33.

#### Remarks.

 The propodeum of the female was described as reddish brown (Momoi 1970). The female specimen, deposited in Osaka Museum of Natural History, Japan, has the apical portion of the propodeum darkish brown and the basal portion brownish black (Figure 12). The specimen, collected from Jiangxi province, China, has the propodeum reddish brown.

#### Specimens examined.

 1 female 1 male, CHINA: Ji’an County, Jiangxi Province, 21 May 2008, leg. Yi Kuang. 1 male, CHINA: Quannan County, 530 m, Jiangxi Province, 28 May 2008, leg. Shi-Chang Li. 1 male, CHINA: Quannan County, 630 m, Jiangxi Province, 7 November 2008, leg. Shi-Chang Li. 18 males, CHINA: Shuangjiang Forest Farm, 174 m, Ji’an County, Jiangxi Province, 25 April to 15 June 2009, leg. Lin-Da Li. 2 males, CHINA: Matubei, 330 m, Quannan County, Jiangxi Province, 27 May 2009, leg. Shi-Chang Li. 3 males, CHINA: Wuyishan, 1170 to 1200 m, Qianshan County, Jiangxi Province, 22 June to 11 July 2009, leg. Zhi-Yu Zhong. 2 males, CHINA: Quannan County, Jiangxi Province, 4 to 11 October 2009, leg. Shi-Chang Li. 1 male, CHINA: Quannan County, Jiangxi Province, 31 May 2010, leg. Shi-Chang Li. 17 males, CHINA: Jiulianshan, 580 m to 680 m, Longnan County, Jiangxi Province, 20 April to 6 June 2011, leg. Mao-Ling Sheng and Shu-Ping Sun. 1 female, CHINA: Shizikou, 200 m to 210 m, Anfu County, Jiangxi Province, 21 June 2011, leg. Zhong-Ping Yu.

**Figures 11–12.**
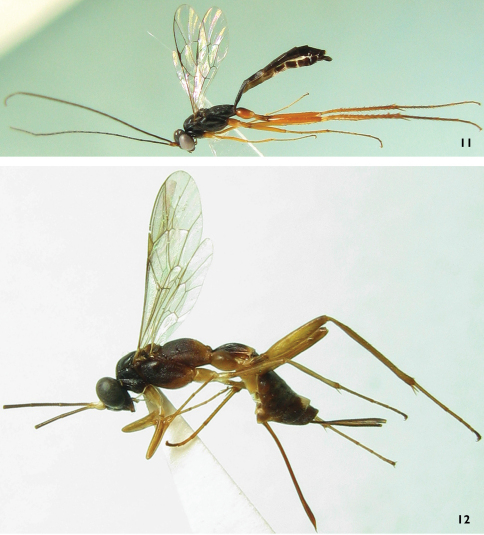
*Ateleute densistriata* (Uchida, 1955) **11** Male body, lateral view **12** Female body, lateral view.

**Figure:**
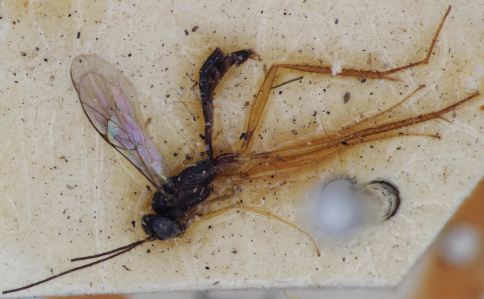
**Figures 13.** *Ateleute spinipes* (Cameron, 1911). Holotype. Male body, lateral view.

## Supplementary Material

XML Treatment for Ateleute

XML Treatment for Ateleute ferruginea

XML Treatment for Ateleute zixiensis

XML Treatment for Ateleute densistriata
